# Distribution, Numbers, and Diversity of ESBL-Producing *E*. *coli* in the Poultry Farm Environment

**DOI:** 10.1371/journal.pone.0135402

**Published:** 2015-08-13

**Authors:** Hetty Blaak, Angela H. A. M. van Hoek, Raditijo A. Hamidjaja, Rozemarijn Q. J. van der Plaats, Lianne Kerkhof-de Heer, Ana Maria de Roda Husman, Franciska M. Schets

**Affiliations:** 1 Centre for Zoonoses and Environmental Microbiology, National Institute for Public Health and the Environment (RIVM), Bilthoven, The Netherlands; 2 Institute for Risk Assessment Sciences, Utrecht University, Utrecht, The Netherlands; Wageningen University and Research Centre, NETHERLANDS

## Abstract

This study aimed to discern the contribution of poultry farms to the contamination of the environment with ESBL-producing *Escherichia coli* and therewith, potentially to the spread of these bacteria to humans and other animals. ESBL-producing *E*. *coli* were detected at all investigated laying hen farms (n = 5) and broiler farms (n = 3) in 65% (46/71) and 81% (57/70) of poultry faeces samples, respectively. They were detected in rinse water and run-off water (21/26; 81%), other farm animals (11/14; 79%), dust (21/35; 60%), surface water adjacent to farms (20/35; 57%), soil (48/87; 55%), on flies (11/73; 15%), and in barn air (2/33; 6%). The highest prevalence and concentrations in the outdoor environment were observed in soil of free-range areas at laying hen farms (100% of samples positive, geometric mean concentration 2.4×10^4^ cfu/kg), and surface waters adjacent to broiler farms during, or shortly after, cleaning between production rounds (91% of samples positive, geometric mean concentration 1.9×10^2^ cfu/l). The diversity of ESBL-producing *E*. *coli* variants with respect to sequence type, phylogenetic group, ESBL-genotype and antibiotic resistance profile was high, especially on broiler farms where on average 16 different variants were detected, and the average Simpson’s Indices of diversity (SID; 1–D) were 0.93 and 0.94 among flock and environmental isolates respectively. At laying hen farms on average nine variants were detected, with SIDs of 0.63 (flock isolates) and 0.77 (environmental isolates). Sixty percent of environmental isolates were identical to flock isolates at the same farm. The highest proportions of ‘flock variants’ were observed in dust (94%), run-off gullies (82%), and barn air (67%), followed by surface water (57%), soil (56%), flies (50%) and other farm animals (35%).The introduction of ESBL-producing *E*. *coli* from poultry farms to the environment may pose a health risk if these bacteria reach places where people may become exposed.

## Introduction

The use of antibiotics in human and animal health care has resulted in the widespread prevalence of antibiotic resistant (ABR) bacteria not only in humans and animals, but also in the environment, e.g. in surface water and soil [1–4]. As a consequence, the probability of getting exposed to ABR bacteria outside a health care setting has increased.

A specific type of antibiotic resistance that currently represents a major public health concern is the 3^rd^ generation cephalosporin resistance induced by extended spectrum beta-lactamase (ESBL)-production [5]. ESBL-producing bacteria are resistant to almost all beta-lactam antibiotics, and often to other classes of antibiotics as well. This results in difficult to treat infections, and additionally compels the use of so-called ‘last-resort antibiotics’, e.g. carbapenems, resulting in increased resistance to these types of antibiotics as well [6]. Initially, ESBL-production was mainly observed in hospital infections caused by *Klebsiella pneumonia*, but today it is also frequently associated with community-acquired infections, mostly urinary tract infections caused by *E*. *coli* [7,8]. In the Netherlands, 1% of urinary tract infections among selected primary care centers in 2009 were associated with ESBL-producing *E*. *coli* [9]; the prevalence of ESBL-producing *E*. *coli* among patients with blood stream infections ranges between 4%- 6% [10]. In community patients and healthy individuals, a prevalence of ESBL-producing Enterobacteriaceae of 5%- 10% has been described [11,12] which, in the study on community patients where species were identified, were shown to be primarily *E*. *coli* [12]. The future threat of increased occurrence of untreatable infections necessitates mitigation of dissemination of ESBL-producing bacteria, and hence the identification of their possible dissemination routes. Spread of ESBL-producing *E*. *coli* in the community may be facilitated by direct contact with human carriers, but alternatively, may also be livestock-related. In the Netherlands, ESBL- producing *E*. *coli* are highly prevalent in poultry: in recent years (2009–2011), ESBL- (and/or AmpC-) producing *E*. *coli* were detected on 100% of Dutch broiler farms studied [13,14]. The high prevalence of ESBL-producing *E*. *coli* on Dutch retail chicken meat, and overlap between ESBL-genotypes from chicken meat and clinical *E*. *coli* isolates, has led to the suggestion of chicken meat as a source of ESBL-producing *E*. *coli* [15,16], although a more recent study demonstrated no evidence for recent events of clonal transmission from poultry to humans using next generation sequencing [17]. The presence of ESBL-producing *E*. *coli* in the environment, including Dutch surface water [18–22], suggests that dissemination through the environment should also be considered.

ABR intestinal bacteria end up in the environment with animal and human faeces. A major human contamination source is wastewater, either discharged onto surface water after treatment by wastewater treatment plants or discharged untreated through sewage overflows during heavy rain fall [13,19,20]. Examples of animal environmental contamination sources are animal manure used for field application and livestock farms [23–28]. At livestock farms, bacteria may enter the natural environment (i.e. ambient air, soil, surface water) directly with droppings of pasture animals and free-range animals, or indirectly from barns, for instance through air and dust, with hands or feet of farm workers, or with rinse water during cleaning practices. Once in the outside environment of farms, the bacteria may spread further away from farms with motile environmental compartments such as air and surface water, where people may get exposed to them, for instance through inhalation of air during outdoor activities, recreation in down-stream located surface water, or when downstream-located water is used for irrigation of crops [18,29,30]. An additional route of dissemination of ESBL-producing *E*.*coli* from farms may be with pest animals, e.g. flies, which have been recognized as transmitters of infectious diseases for some time [31]. Flies may move from farms where they were bred in, and have fed on, faeces and carcasses [32], to next feed on food meant for human consumption, including prepared food. Indeed, carriage of ESBL-producing *E*. *coli* by flies on livestock farms has been described previously [23,33,34].

The current study was aimed at determining the extent of contamination of the natural (or outside) environment at Dutch poultry farms with ESBL-producing *E*. *coli*. For this purpose, ESBL-producing *E*. *coli* were quantified in ambient air, soil, surface water and flies at laying hen and broiler farms. For comparison, the bacteria were simultaneously quantified in the suspected sources of contamination of the natural environment, e.g. poultry faeces, wastewater, barn dust and barn air. Additionally, isolates from different matrices were characterised with respect to ESBL-genotype, antibiotic resistance profile, phylogenetic group, and sequence type, to determine the proportion of environmental isolates identical to those observed in poultry. The current study strengthens the knowledge base on the contribution of animal husbandry to the dissemination of ESBL-producing bacteria to the environment and therewith, to the potential spread of ABR zoonoses that cause hard to treat human infections.

## Material and Methods

### Ethics Statement

The field samples were obtained at private properties, permission was granted by the collaborating farmers.

### Poultry Farms

**Fig 1:**
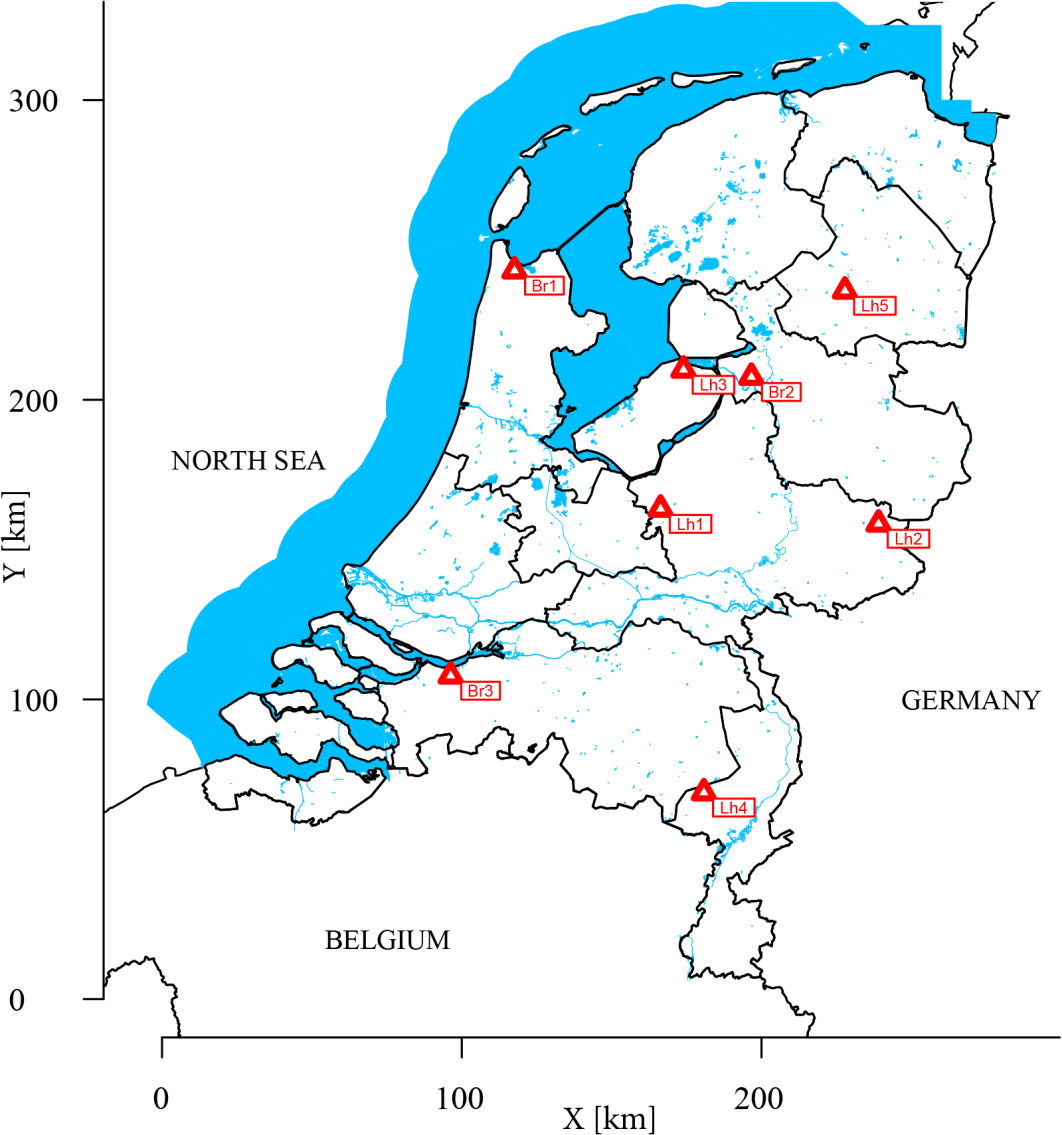
Map of the Netherlands showing locations of the sampled poultry farms.

During 2011 and 2012 three broiler (Br1 to Br3) and five laying hen (Lh1 to Lh5) farms were visited multiple times (Fig 1). Each first visit had an orienting character, during which farms and their immediate surroundings were mapped, suitable sites for sampling were identified, and poultry faeces was sampled to establish the presence or absence of ESBL-producing *E*. *coli*. After this, laying hen farms were visited once more during the same production round, and broiler farms were visited during a subsequent production round: once while broilers were present, and once just after these broilers were removed and stables had just been cleaned or were being cleaned. Sampling strategies were different between farm types due to the high turnover of broiler flocks (every six to seven weeks) and the low turnover of laying hens (approximately once in every one and a half year). Farm Br1 was sampled during three different production rounds, two in 2011 and one in 2012, i.e. in total on six occasions after the first orientating visit. All broiler farms were conventional farms, with capacities of 38,000 (Br3), 87,000 (Br1) and 150,000 (Br2) broilers. Two of the laying hen farms were conventional farms with capacities of 78,000 (Lh2) and 80,000 (Lh4) chickens, and the three other ones were free range farms with capacities of 30,000 (Lh1, Lh5) and 43,000 (Lh3) chickens. During the main sampling occasions (i.e. after the orienting visit), the age of broiler flocks was four to five weeks, the age of laying hen flocks was on average 59 weeks (range 26–73 weeks).

### Description of Sampled Matrices

Overall, 471 samples were taken, 276 from broiler farms and 195 from laying hen farms: 290 (62%) from environmental compartments (surface water, soil, dust, air, flies), 141 (30%) from poultry faeces, 26 (5.5%) from wastewater, 14 (3.0%) from faeces from other animals kept at the farms (S1 Table). Fresh (i.e. still soft and warm) and semi-fresh (i.e. generally still identifiable as individual droppings, but no longer soft and warm) poultry faeces was sampled from barns and free-range areas. At laying hen farms, poultry faeces was additionally sampled from manure belts and at different stages during air-drying. At broiler farms, when present, faeces was additionally sampled from dung-heaps. The reason for additional sampling of faeces from manure belts, during various stages of air drying and from dung-heaps was that it was anticipated that bacterial concentrations might change after prolonged time outside the body. Soil was sampled in free-range areas where applicable, and from various other sites at the premises, i.e. in the vicinity (1–5 m) of barns, manure storage sheds, wastewater storage basins, or manure belts, as well as at sites not visibly under direct influence of such faecal contamination sources. At three of the farms, soil samples were taken from dried-up ditches. When present, surface water was sampled from ditches bordering on, or within 50 m distance of farm premises, as well as from more remote (50 m– 1 km) water bodies or sampling sites. Wastewater was sampled at broiler farms from drains, collection pits, storage basins and run-off gullies. Wastewater in drains, pits and basins consisted of barn rinse water (either or not diluted with rain water), and was therefore considered to reflect poultry faeces contents. This in contrast to water from run-off gullies, which consisted of run-off from farm premises and might therefore contain input from other, undefined contamination sources. Sometimes, the water level in run-off gullies was low, and sediment was sampled instead. Only one of the laying hen farms had a wastewater pit filled with wastewater at the time of sampling, and this was sampled as well. Other farm animals included hobby laying hens at broiler farms (Br1, Br2), a dog (Br1), cattle (Br2, Lh2), horses (Lh3), and swallows (Lh2). Flies were caught inside barns, from other indoor environments (manure storage sheds, egg-sorting areas, homes, break rooms or changing rooms, stables of other animals) and outdoors. Caught flies included *Musca domesticus* (common house fly), *Stomoxys calcitrans* (stable fly), *Fannia canicularis* (lesser housefly), *Calliphoridae* (blowflies), Hydrotaea, *Muscina stabulans* (false stable fly), *Sarcophagidae* (flesh flies) and *Tachinidae* (tachina flies). Dust was sampled from surfaces inside barns, from barn ventilation fans, from a manure storage shed, and, at Br1, outside one of the barns from a fence positioned within 5 m range of a ventilation fan. Most air samples were collected inside barns. At Br1 outdoor air was also sampled (within 5 m of ventilator fans) as well as air from a break room adjoining one of the barns. All samples were transported to the laboratory and stored at 5±3°C (except for flies which were stored at room temperature), and analysed within 24 hours after sampling. The procedures of sample collection and subsequent preparation is described in S1 Materials and Methods).

### Isolation and Enumeration of *E*. *coli* and ESBL-Producing *E*. *coli*

Multiple dilutions of each sample were streaked on ChromID ESBL agar (Biomerieux, Boxtel, the Netherlands) for the isolation of ESBL-producing *E*. *coli* and on tryptone bile x-glucuronide agar (TBX) for the isolation of *E*. *coli*, in accordance with ISO 16649–2 [35] (for details on sample preparation, see S1 Material and Methods). For water, samples were filtered through 0.45 μm pore size membrane filters, which were next placed on these media. Cultures were incubated for 4 to 5 hours at 36±1°C, followed by 21±3 h at 44±0.5°C[35]. For the isolation of ESBL-producing *E*. *coli*, environmental samples (with the exception of surface water) were additionally pre-enriched in buffered peptone water supplemented with 1 μg/ml cefotaxime (CTX), using the same incubation conditions. Pre-enriched suspensions were streaked onto ChromID ESBL agar and incubated for another 21±3 h at 44±0.5°C. Bacterial concentrations were calculated from the colony counts obtained from the direct cultures using Mathematica software 9.0.1 (WolframResearch, Champaign, IL, USA). The pre-enriched cultures were assessed for presence or absence of suspected colonies, which was used as supplementary data in case of negative direct cultures. Because of the skewed distribution of concentrations of ESBL-producing *E*. *coli* within matrices and the relatively high numbers of negative samples for most environmental matrices, group mean values were expressed as geometric means (i.e. based on the log-transformed concentrations). Samples yielding ESBL-producing *E*. *coli* after enrichment but not in direct culture were included for estimation of the geometric means, by setting the concentration in these samples to $\frac{1}{x}$ cfu/g, in which *x* is the amount of matrix that was cultured.

### Phenotypic Confirmation of ESBL-Production

Suspected ESBL-producing *E*. *coli* isolates (n = 1,140) were confirmed to be indole-positive using BBL Dry Slide (BD), and subsequently tested for ESBL-production by disk diffusion following CLSI guidelines [36], using Sensi-Discs (BD, Breda, the Netherlands). Zone diameters were determined for cefotaxime (30μg) ± clavulanic acid (10μg), ceftazidime (30μg) ± clavulanic acid (10μg), and cefoxitin (30 μg). ESBL-producing isolates were defined as strains resistant to cefotaxime (zone diameter ≤ 22 mm) and/or ceftazidime (zone diameter ≤ 17 mm), and an increase in zone diameter of ≥ 5 mm with the disks containing clavulanic acid [36]. ESBL-producing *E*. *coli* concentrations were calculated from the numbers of β-glucuronidase-positive colonies, and the fraction of isolates confirmed to be indole-positive and ESBL-producing. Some isolates (n = 22) did not appear to be resistant to either cefotaxime or ceftazidime, and then ESBL-production was confirmed using an alternative AmpC and ESBL detection test, which is based on cefpodoxime (Mastgroup Ltd., Bootle, UK). Third-generation cephalosporin-resistant isolates with no significant inhibitory effect of clavulanic acid (as defined by CLSI) were defined as non-ESBL-producers and excluded from further analyses (n = 32).

### Phylogenetic Group Analysis

Of each isolate, material from one single colony was suspended in Tris EDTA buffer (pH 8.0, Sigma-Aldrich, Zwijndrecht, the Netherlands), followed by incubation at 99°C for 5 min. The resulting cell lysates were stored at -20°C. PCRs were targeted to *chuA* and *yjaA* genes, and TspE4.C2 DNA fragment using primers and PCR conditions as described by Clermont at al. [37]. Strains were sub-grouped according to Escobar-Páramo et al. [38]: subgroup A_0_, chuA-, yjaA-, TspE4.C2-; subgroup A_1_, chuA-, yjaA+, TspE4.C2-; group B1, chuA-, yjaA-, TspE4.C2+; subgroup B2_2_, chuA+, yjaA+, TspE4.C2-; subgroup B2_3_, chuA+, yjaA+, TspE4.C2+; subgroup D_1_, chuA+, yjaA-, TspE4.C2-; subgroup D_2_, chuA+, yjaA-, TspE4.C2+. To confirm the *E*. *coli* identity of A_0_ isolates, a PCR was performed targeted to the β-glucuronidase gene *uidA* [39]. UidA-negative isolates (n = 1) were excluded from further analysis.

### ESBL-Genotyping

The presence of CTX-M-group 1, CTX-M-group 2, CTX-M-group 9, and OXA-, SHV- and TEM-genes was determined by multiplex PCR using primers and PCR conditions described by Dallenne et al. [40]. The ESBL-genotypes were based on partial gene sequences. For this purpose, PCR-products of the expected size were treated with ExoSAP-IT (GE Healthcare, Hoevelaken, the Netherlands) and sequenced using the same primers used to generate the PCR-products. To confirm the results based on partial gene sequences, 54% of the major genotypes CTX-M-1, SHV-12 and TEM-52, was sequenced full-length. For this purpose, DNA of the selected isolates was subjected to PCR analysis using primers and conditions described by Dierikx et al. [41]. Obtained sequences were compared with ESBL-gene sequences in the GenBank database and on the Lahey website (www.lahey.org/Studies).

### Multilocus Sequence Typing (MLST)

Seven house-keeping genes, *adk*, *fumC*, *gyrB*, *icd*, *mdh*, *purA* and *recA*, were amplified and sequenced as described by Wirth et al.[42]. Primer sequences were obtained from the *E*. *coli* MLST database website http://mlst.warwick.ac.uk/mlst/mlst/dbs/Ecoli and used at 0.2 μM per reaction. Amplification conditions were: 15 min 95°C, followed by 35 cycles of 30s 95°C, 30s 65°C (*adk*, *fumC*, *gyrB*, *icd*, *purA*, *recA*) or 30s 60°C (*mdh*), 45s 72°C, and a final elongation step of 10 min 72°C. PCR-products were analysed on agarose gel and PCR-products of the expected size were treated with ExoSAP-IT (GE Healthcare, Hoevelaken, the Netherlands) followed by sequencing using the same primers used to generate PCR-products. Sequence types were identified against the online database available at the *E*. *coli* MLST website (http://mlst.warwick.ac.uk/mlst/mlst/dbs/Ecoli) using Bionumerics software (version 7.1; Applied Maths NV, Sint Martens-Latem, Belgium). The same software was used for the construction of minimal spanning trees based on the concatenated sequences of the seven alleles.

### Antibiotic Resistance Profiling

Isolates were screened for antibiotic susceptibility to a panel of 14 antibiotics of human and veterinary clinical relevance, using micro broth dilution using the Sensititre SensiTouch system (MCS Diagnostics, Swalmen, the Netherlands), according to the manufacturers’ instructions and CLSI guidelines [36]. Included were one or two antibiotic representatives from seven classes of antibiotics: ampicillin and co-amoxiclav (penicillins), cefotaxime and ceftazidime (3rd generation cephalosporins), tetracycline (tetracyclines), ciprofloxacin and nalidixic acid ((fluoro)quinolones), gentamycin and streptomycin (aminoglycosides), sulfamethoxazole and trimethoprim (folate pathway inhibitors), chloramphenicol (phenicols), and imipenem and meropenem (carbapenems). Resistance was defined as having a minimal inhibitory concentration (MIC) above the ecological cut-off (ECOFF) value available at the EUCAST website [43]. For amoxicillin/clavulanic acid a break-point of ≥16/8 was used [44], because an ECOFF value was not available at the EUCAST website at the time of data analysis. Multi-drug resistance was defined as resistance to 3 or more different classes of antibiotics [45].

### Selection of Isolates

Overall, 1,107 confirmed ESBL-producing *E*. *coli* isolates were obtained from 240 positive samples. Per sample, a maximum of two isolates with similar ESBL-phenotype based on inhibition zones and the effect of clavulanic acid for cefotaxime, ceftazidime and, if applicable, cefpodoxime, were included for further analyses. The resulting 686 isolates were analysed with respect to phylogenetic group. Subsequently, maximally two isolates with similar ESBL-phenotype and identical phylogenetic group per sample (or set of samples derived from the exact same site at the same time), were characterized at the level of ESBL-gene family using PCR (n = 488, from 230 samples). These data were used to identify ‘sets’ of isolates, i.e. isolates obtained from different matrices at the same farm, with the same identity with respect to phylogenetic group and ESBL-gene family (n = 434), as well as ‘unique variants’, i.e. variants that were detected in only one of the matrices (n = 54). Within each set of isolates, at least one isolate per matrix (n = 275), as well as one of each unique variant (n = 41) were further characterized with respect to ESBL-genotype and antibiotic resistance profiles (n = 316). Eighteen of the 316 isolates were retrospectively identified as ‘copy-isolates’, i.e. isolates with the same characteristics obtained from the same sample; these were excluded from data analyses, leaving 298 isolates: 115 from 87 faeces and rinse water samples, and 183 from 114 environmental samples (Table 1). Of these, 288 were characterized using MLST (excluded from MLST analysis were 10 of 28 faeces isolates that, with respect to phylogenetic group, ESBL-genotype and ABR profile, had no counterparts in any of the environmental samples).

**Table 1:** Numbers of characterized ESBL-producing *E*. *coli* isolates per matrix and farm type.

Matrix	Laying hens	Broilers	Total
***Faeces and wastewater***			
**Poultry faeces** *	41 (4)	52 (6)	93
**Rinse water from barns**	1	21	22
***Farm environment***			
**Air**	0	3	3
**Dust**	5	12	17
**Faeces of other animals**	4	13	17
**Flies**	7	7	14
**Run-off gullies**	0	17	17
**Soil**	38	24	62
**Surface water**	11	42	53
***Total***	***107***	***191***	***298***

* Indicated between brackets are the numbers of isolates that were not characterized using MLST.

### Statistics

The Pearson Chi square Test was used to test differences in the proportions of positive samples (e.g. prevalence in adjacent vs. remote surface waters, prevalence in soil at laying hen and broiler farms), and differences in the proportions of isolates identical to flock isolates between broiler and laying hen farms. Non-parametric tests were used to test for differences in bacterial concentrations between groups. The rationale for this was the skewed distribution of ESBL-producing *E*. *coli* concentrations, the inclusion of semi-quantitative pre-enrichment data (because of which also log-transformed values were not always normally distributed), and the loss of information when excluding negative samples from analysis, as is the case when using log-transformed values. The Mann-Whitney U Test and the Kruskal-Wallis Test were used for comparison between two and multiple groups, respectively.

To express the diversity of ESBL-producing *E*. *coli* variants in laying hen and broiler farms, the Simpson’s Index of Diversity (1-D) was calculated for poultry faeces and barn rinse water isolates, for each farm. The Simpson’s Index of Diversity (1-D) was calculated from:

$$D=\frac{\sum_{i=1}^{N}n_{i}\;(n_{i}-1)}{N\;(N-1)}$$

where n_i_ represents the number of variants with the i^th^ pheno-/genotype, and N the total number of isolates. Br1 was sampled during three different production rounds, yielding three slightly different diversity indices, which were averaged for purpose of statistical analysis. The difference in diversity indices between laying hen and broiler farms was tested using the Mann-Whitney U test, the difference between faeces and environmental samples was tested in a paired fashion using the Wilcoxon Signed Ranks Test (non-parametric tests were used because of low numbers of farms).

## Results

### Prevalence of ESBL-Producing *E*. *coli* at Poultry Farms

**Fig 2:**
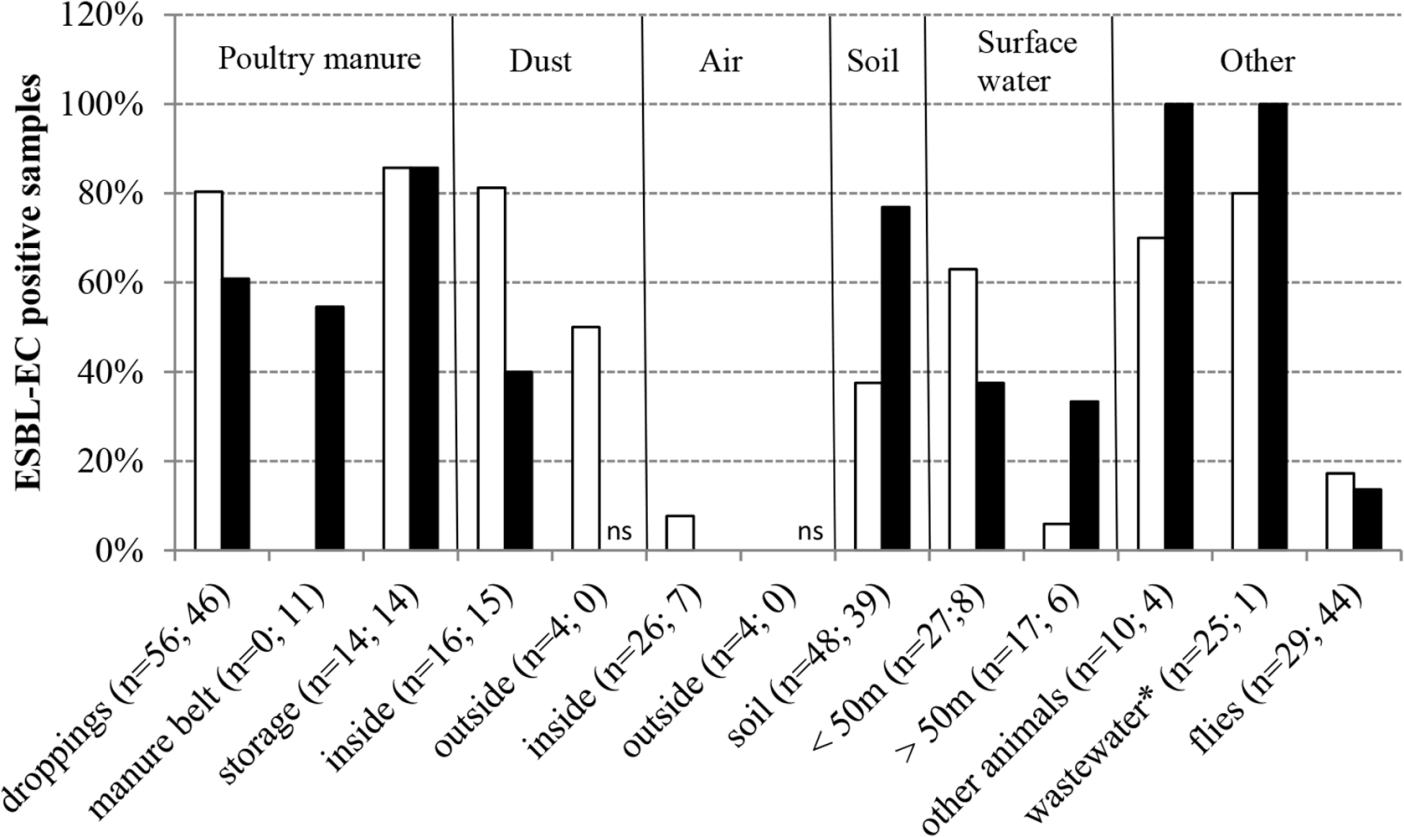
Prevalence of ESBL-producing *E*. *coli* in poultry faeces and environmental samples at laying hen and broiler farms. White bars represent broiler farms, black bars represent laying hen farms. In between brackets (n = a; b) are indicated the numbers of analysed samples at broiler (a) and laying hen farms (b) respectively; ns = not sampled; *includes water from pits and storage basins as well as run-off water and sediment from gullies.

ESBL-producing *E*. *coli* were present at each poultry farm, in 65% and 81% of poultry faeces samples at laying hen and broiler farms, respectively (Fig 2). The bacteria were also highly prevalent in wastewater and sediments from run-off gullies, in faeces from other animals present at the farms, in dust in the interior of barns at broiler farms, and in soil at laying hen farms. Also surface water in the direct vicinity of the farms frequently contained ESBL-producing *E*. *coli*, and so did, to a lesser extent, flies and barn air (Fig 2).

At laying hen farms, ESBL-producing *E*. *coli* were less frequently detected in dust and air in barns (40% and 0% resp.) than at the broiler farms (81% and 7.7% resp.). Overall, ESBL-producing *E*. *coli* were detected in two of 33 indoor air samples which were both obtained at the same broiler farm (Br1), at two different sampling points. At the same broiler farm, dust and air were also sampled near ventilator exhausts outside barns; ESBL-producing *E*. *coli* were detected in 50% of the outside dust but none of the outside air samples.

In the outdoor environment, ESBL-producing *E*. *coli* were most frequently detected in soil at laying hen farms (77%), followed by surface water adjacent to broiler farms (63%). The prevalence in soil was higher at laying hen farms compared to broiler farms (*P*<0.0001), while the prevalence in adjacent surface water was higher at broiler farms, although this latter difference was not statistically significant (*P*>0.1) (Fig 2). The prevalence of ESBL-producing *E*. *coli* in surface waters adjacent to broiler farms was ten times higher than that at more remote sampling sites (*P*<0.0001). Moreover, in adjacent surface waters, the bacteria were detected more frequently during or shortly after cleaning compared to when broilers were present (91% vs. 44% of samples positive; *P* = 0.01). In surface waters adjacent to laying hen farms, the prevalence of ESBL-producing *E*. *coli* was similar to that at more remote sampling sites (*P*>0.1) (Fig 2).

ESBL-producing *E*. *coli* were detected in 14% and 17% of all fly samples at laying hen and broiler farms, respectively (Fig 2). All ESBL-positive flies belonged to four of the eight different fly species/families that were caught at the farms: blow flies (*Calliphoridae*), common house flies (*Musca domestica*), stable flies (*Stomoxys calcitrans*) and flesh flies (*Sarcophagidae)*. Among the 52 pooled samples that consisted of flies from these four species/families, the overall prevalence was 21%. Total *E*. *coli* were detected in 85% of these samples. Although ESBL-producers were not detected in samples consisting of the other four fly species/families (*Fannia canicularis*, *Hydrotea*, *Muscina stabulans*, *Tachinidae*), these flies did carry *E*. *coli* (53%). For the most abundant fly species that were caught at both types of farms, *M*. *domestica* (n = 32 pools) and *S*. *calcitrans* (n = 11 pools), a two-fold higher prevalence of ESBL-producing *E*. *coli* was observed among flies from broiler farms compared to flies from laying hen farms: 33% vs. 16% ESBL-positive, respectively (*P*>0.1).

### Concentrations of ESBL-*E*. *coli* and *E*. *coli* in Poultry Farm Environment

Concentrations of ESBL-producing *E*. *coli* and total *E*. *coli* were determined in poultry faeces, wastewater, soil, surface water, flies, and a subset of dust samples (Table 2). In fresh and semi-fresh droppings collected in barns and free-range areas (the latter being only applicable for Lh1, Lh3, and Lh5), the concentrations of ESBL-producing *E*. *coli* were approximately 2-log higher on broiler farms compared to laying hens farms (geometric means 5.0×10^5^ and 2.3×10^7^ cfu/kg, respectively; *P =* 0.001). The proportion of ESBL-producing *E*. *coli* was slightly higher among broilers compared to laying hens: on average one in 3.1×10^3^ *E*. *coli* were ESBL-producing, compared to one in 1.8×10^4^, respectively, although this difference was not statistically significant (*P*>0.1). Five barn dust samples, three at broiler farm Br1 and one each at laying hen farms Lh4 and Lh5, were analysed quantitatively. Concentrations of ESBL-producing *E*. *coli* and total *E*. *coli* were 1- to 3-log_10_ units lower than that in poultry faeces from barns (Table 2).

**Table 2:** ESBL-producing *E*. *coli* and *E*. *coli* concentrations in poultry manure and the environment.

Matrix (concentration unit).and matrix subgroups (no. samples^a^)	ESBL-producing *E*. *coli*			*E*. *coli*		
	% pos^b^	Geo- mean	Range^c^	% pos^b^	Geo- mean	Range^c^
**Poultry faeces (cfu/kg)**						
Laying hen_barns (n = 18)	56	6.9×10^5^	≥10^2^–1.7×10^9^	100	7.8×10^9^	1.4×10^9^–5.5×10^10^
Laying hen_ree range (n = 9)	78	3.2×10^5^	≥10^2^–1.2×10^7^	100	1.4×10^10^	4.5×10^9^–3.7×10^7^
Laying hen_ manure belts (n = 9)	56	1.4×10^6^	3.1×10^4^–4.5×10^7^	100	9.0×10^10^	1.7×10^10^–1.8×10^12^
Laying hen_ storage containers (n = 14)	88	4.4×10^6^	≥10^2^–1.0×10^9^	94	1.6×10^11^	1.2×10^9^–1.8×10^12^
Broiler_ barns* (n = 36;28)	83	2.3×10^7^	≥10^2^–8.3×10^9^	100	4.4×10^10^	1.4×10^9^–2.2×10^11^
Broiler_dung heaps (n = 14)	93	2.5×10^3^	≥10^2^–7.1×10^6^	86	2.5×10^7^	2.7×10^5^–1.8×10^9^
**Wastewater (cfu/l)**						
Pits/basins/drains at cleaning* (n = 15;10)	80	2.4×10^6^	9.0×10^3^–8.3×10^7^	80	3.2×10^8^	1.3×10^5^–4.6×10^9^
Run-off gullies* (n = 5;4)	80	7.8×10^3^	4.3×10^2^–2.2×10^5^	100	1.5×10^5^	3.6×10^3^–6.0×10^6^
**Wastewater (cfu/kg)**						
Sediment from run-off gullies* (n = 4;2)	100	2.4×10^5^	4.5×10^5^–1.0×10^6^	100	1.5×10^7^	1.2×10^7^–2.0×10^7^
**Dust (cfu/kg)**						
Laying hen_barns (n = 2)	50	≥2×10^2^	n.a.	100	4.1×10^8^	1.3×10^8^–1.4×10^9^
Broiler_barns (n = 3)	100	5.0×10^5^	2.5×10^5^–8.8×10^5^	100	6.4×10^7^	5.7×10^7^–7.7×10^7^
**Soil (cfu/kg)**						
Free-range (n = 14)	100	2.4×10^4^	≥10^2^–3.3×10^7^	100	1.1×10^9^	1.8×10^7^–5.4×10^9^
Near barns* (n = 23;21)	43	1.7×10^2^	≥10^2^–2.0×10^4^	100	7.5×10^4^	8.2×10^3^–2.6×10^6^
Near manure storage* (n = 19;18)	68	4.4×10^2^	≥10^2^–2.0×10^4^	100	1.2×10^5^	≥10^2^–1.8×10^8^
Near rinse water (n = 3)	67	1.1×10^4^	≥10^2^–1.2×10^6^	100	1.1×10^5^	≥10^2^–6.8×10^7^
Dried-up ditches* (n = 11;10)	45	3.8×10^2^	≥10^2^–7.8×10^4^	100	6.9×10^5^	9.0×10^3^–1.7×10^8^
Premises other* (n = 17;15)	24	3.2×10^2^	≥10^2^–1.0×10^4^	100	1.1×10^4^	≥10^2^–1.4×10^6^
**Surface water (cfu/l)**						
At > 50m distance (n = 23)	13	4	1.4–10	100	1.8×10^3^	31–8.0×10^4^
Laying hen, < 50m (n = 8)	38	27	11–1.4×10^2^	100	5.0×10^3^	90–1.4×10^6^
Broiler_flocks present *, < 50m (n = 16;13)	50	25	1.4–2.3×10^3^	100	3.6×10^3^	3.1×10^2^–1.2×10^5^
Broiler_at cleaning, <50m (n = 11)	91	1.9×10^2^	2.8–5.0×10^5^	100	2.5×10^4^	6.3×10^2^–3.1×10^7^
**Flies (cfu/fly pool)** ^d^						
*Calliphoridae* (n = 8, 20)	13	2.5×10^4^	n.a.	50	6.3×10^2^	≥3.3–2.4×10^5^
*Musca domestica* (n = 32; 212)	22	5.2×10^2^	≥3.3–8.0×10^4^	97	3.0×10^5^	≥3.3–1.2×10^8^
*Sarcophagidae* (n = 1; 2)	100	4.1×10^3^	n.a.	100	5.1×10^8^	n.a.
*Stomoxys calcitrans* (n = 11; 39)	18	1.5×10^3^	1.2×10^3^–2.0×10^3^	82	9.4×10^2^	≥3.3–5.9×10^7^
Other (n = 21; 53)	0	0	n.a.	53	1.6×10^4^	≥3.3–1.5×10^8^

^a^For some of the samples total *E*. *coli* was not determined (*): in these cases the number of samples analyzed for ESBL-producing *E*. *coli* (a) and *E*. *coli* (b) are indicated separately (n = a; b).

^b^Percentages of samples positive for the indicated bacteria.

^c^ Concentration ranges observed in the positive samples

^d^ Indicated after fly species/family (n = x, y) is the number of pools (x) and the number of flies (y)

cfu = colony forming units; Geo-mean = geometric mean; n.a. = not applicable.

In soil, the concentrations of ESBL-producing *E*. *coli* were significantly higher in free-range areas at laying hen farms compared to other sites at laying hen and broiler farms (geometric means 2.4×10^4^ vs. 3.6×10^2^ and 3.9×10^2^ cfu/kg, respectively; *P* = 0.001). A relative high concentration was also observed at sites that were visibly influenced by rinse water (1.1×10^4^ cfu/kg), although this value is based on only two (out of three) positive samples with substantially different concentrations (Table 2). In surface water bodies adjacent to poultry farms, the concentrations of ESBL-producing *E*. *coli* were higher than in further removed water bodies (geometric means 66 and 3.9 cfu/l, respectively; *P*<0.0001). Moreover, at broiler farms, ESBL-producing *E*. *coli* were present in higher concentrations during or shortly after cleaning compared to when broilers were present (geometric mean of 1.9×10^2^ vs. 25 cfu/l, respectively; *P* = 0.02). An explanation might be contamination of surface water with rinse water, given the high concentrations of ESBL-producing *E*. *coli* in rinse water (geometric mean of 2.4×10^6^ cfu/l). No significant difference in ESBL-producing *E*. *coli* concentrations was observed between surface waters surrounding laying hen farms and broiler farms when flocks were present at both types of farms (Table 2).

In fly samples containing flies from the four species/families shown to carry ESBL-producing *E*. *coli*, the geometric mean concentrations of ESBL-producing *E*. *coli* and total *E*. *coli* was 1.1×10^3^ cfu/pool and 6.3×10^4^ cfu/pool, respectively. Even though ESBL-producing *E*. *coli* were not detected in the other four species/families, the *E*. *coli* concentrations in these fly samples were in the same order of magnitude: 1.6×10^4^ cfu/pool) (Table 2).

### Diversity among ESBL-Producing *E*. *coli* at Poultry Farms

**Fig 3:**
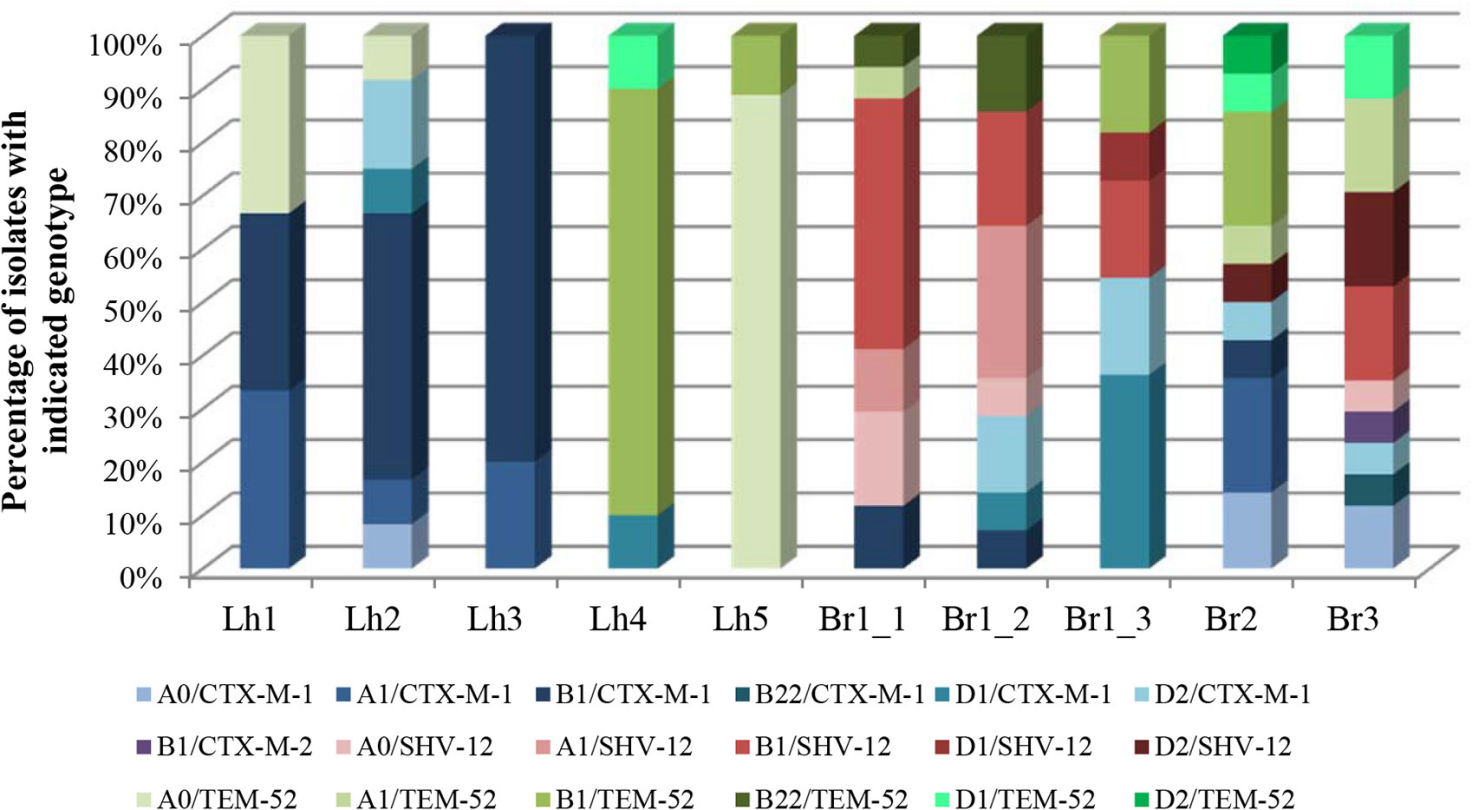
Diversity among ESBL-producing *E*. *coli* variants in poultry faeces and barn rinse water. Shown are the proportions of ESBL-producing isolates with the indicated phylogenetic group and ESBL-genotype combinations, among the isolates from poultry faeces and barn rinse water sampled from drains and pits. Lh = laying hen farms, Br = broiler farms.

Among 298 isolates from poultry faeces and farm environment, all phylogenetic subgroups were represented, the most prevalent being subgroup B1 (42%), followed by A_1_ (24%), A_0_ (11%), D_2_ (10%), D_1_ (7.7%), B2_2_ (4.7%) and B2_3_ (0.7%). The vast majority (98%) of all isolates carried *bla*_CTX-M-1_ (41%), *bla*_SHV-12_ (29%) and *bla*_TEM-52_ (28%). Additional detected genotypes were *bla*_CTX-M-2_ (0.34%), *bla*_CTX-M-14_ (0.34%), *bla*_CTX-M-15_ (0.34%), and *bla*_CTX-M-27_ (0.34%). Two isolates (0.67%) contained an unidentified ESBL-gene other than *bla*_CTX-M_, *bla*_TEM_, *bla*_SHV_, and *bla*_OXA_. In total, 25 different variants were observed based on phylogenetic subgroup and ESBL-genotype. Eighteen of these were found in poultry faeces and rinse water from barns, and therewith considered to reflect the population of the flocks (Fig 3). With the exception of one of the isolates from broiler faeces that carried *bla*_CTX-M-2,_ all faeces and rinse water isolates (99%) carried *bla*_CTX-M-1_, *bla*_SHV-12_ or *bla*_TEM-52_. The distribution of these three genotypes differed between the two farm types: *bla*_CTX-M-1_was present in 32% and 50% of the isolates on broiler and laying hen farms respectively (*P* = 0.049), *bla*_SHV-12_ in 45% compared to 0% (*P<*0.0001), and *bla*_TEM-52_ in 22% and 50% of the isolates (*P* = 0.002). The diversity among ESBL-producing *E*. *coli* from poultry faeces and rinse water with respect to phylogenetic subgroup and ESBL-genotype combination was higher on broiler farms compared to laying hen farms (Fig 3). This was confirmed by the respective average Simpson’s Indices of Diversity (SID) of 0.89 and 0.51 (*P* = 0.04, Table 3).

**Table 3:** Simpson’s Index of Diversity (1-D) among isolates from poultry faeces and farm environment.

		Average Simpson’s Index of Diversity [range]	
Origin of isolates^a^	Isolate characteristics^b^	Broilers (n = 3)	Laying hens (n = 5)
**Flocks**	Phylo/ESBL	0,89 [0.82–0.92]	0,51 [0.22–0.76]*
**Flocks**	ST/Phylo/ESBL/ABR	0.93 [0.91–0.96]	0,63 [0.22–0.92]
**Flocks**	ESBL/ABR	0,90 [0.83–0.93]	0,37 [0.00–0.56]*
**Environment**	Phylo/ESBL	0,88 [0.83–0.92]	0,60 [0.40–0.90]
**Environment**	ST/Phylo/ESBL/ABR	0,94 [0.92–0.94]	0,77 [0.44–1.00]°
**Environment**	ESBL/ABR	0,91 [0.88–0.93]	0,57 [0.17–0.90]°

^a^ Isolates from faeces and rinse water (‘Flocks) or isolates from the farm environmental (‘Environment’)

^b^ Diversity was analysed for three combinations of isolate characteristics: phylogenetic subgroup and ESBL-genotype; (Phylo/ESBL); ST, phylogenetic subgroup, ESBL-genotype and ABR profile (ST/Phylo/ESBL/ABR) and ESBL-genotype and ABR profile (ESBL/ABR)

* = At the same characteristics level, the difference between broiler and laying hen farms is statistically significant (*P*<0.05) using the Mann-Whitney U test

° = Within farm type and at the same characteristics level, the difference between ‘Flocks’ and ‘Environment’ is statistically significant (*P*<0.05) using the Wilcoxon Signed Ranks test.

Overall, 65 different ESBL-producing *E*. *coli* sequence types (STs) were detected at poultry farms: 45 at broiler farms, of which 27 (60%) in poultry faeces and rinse water, and 29 at laying hen farms, of which ten (34%) in poultry faeces and rinse water. Among these were seven new STs (ST4976 –ST4981, ST4994). Thirteen (20%) of the STs were observed at multiple farms, and nine of these were detected at both types of farms (ST10, ST48, ST58, ST155, ST162, ST212, ST746, ST1276, ST3249). The most widespread STs were ST48 and ST155, which were both detected at six of the eight farms.

**Fig 4:**
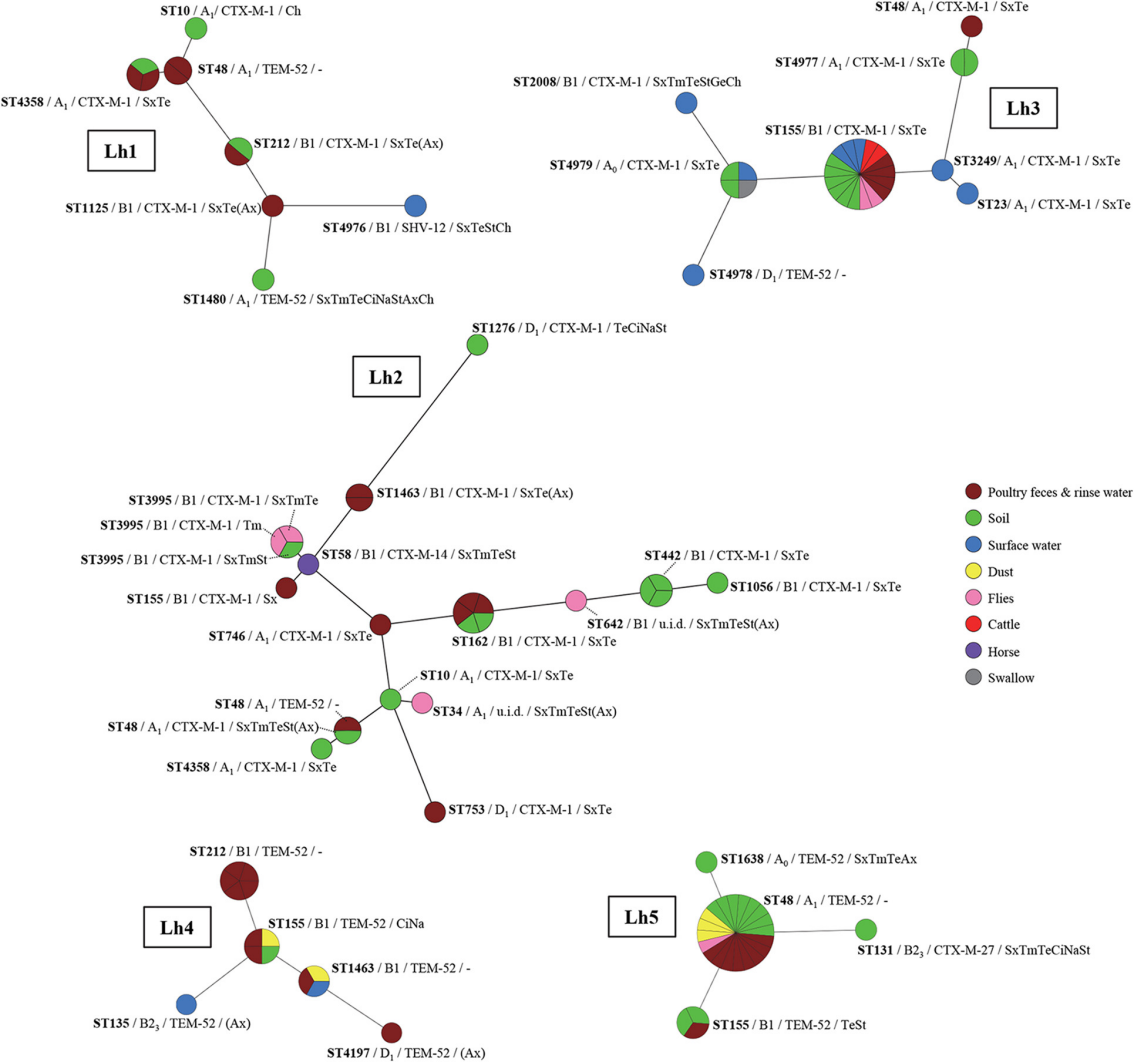
ESBL-producing *E*. *coli* variants on laying hen farms. Maximum parsimony trees constructed based on the concatenated sequences of the seven MLST alleles, using Bionumerics 7.1 software. Node sizes reflect the number of isolates per ST and node colours represent different matrices. Additionally indicated are the phylogenetic group, ESBL-genotype and ABR profiles of isolates in every node (i.e., ST). Note that per sample maximally one isolate of each variant was included, and that multiple isolates of a specific variant reflects detection in multiple samples. ABR profiles represent antibiotics to which resistance was observed additionally to 3rd generation cephalosporins. In between brackets are antibiotics with MICs just above and just below epidemiological cut-off values (i.e. with a maximal 2-fold difference) among isolates with the same ST and/or ESBL genotype. Sx = sulfamethoxazole, Tm = trimethoprim; Te = tetracycline, Ci = ciprofloxacin, Na = nalidixic acid, St = streptomycin, Ge = gentamycin, Ax = amoxicillin+clavulanic acid, Ch = chloramphenicol; u.i.d. = unidentified.

**Fig 5:**
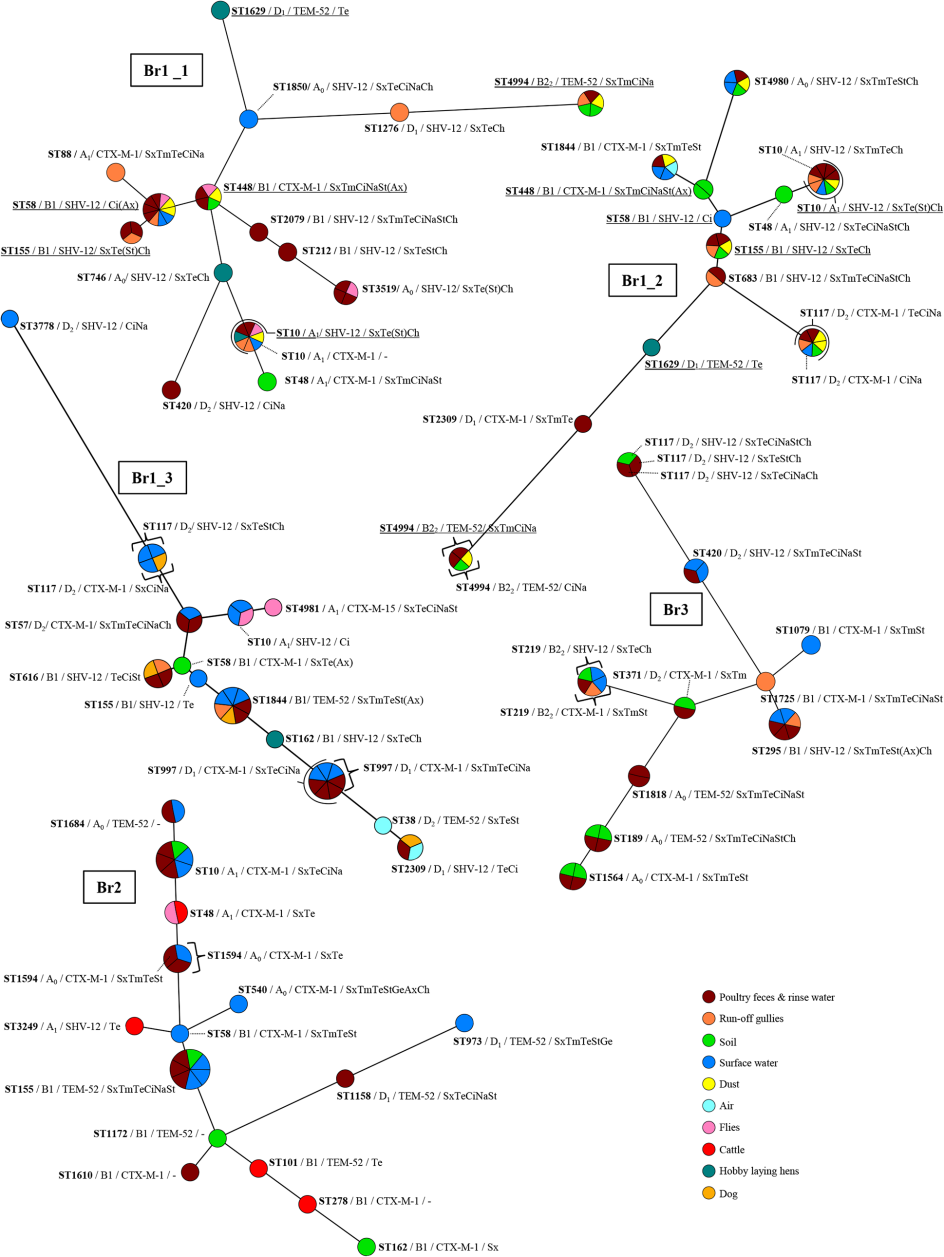
ESBL-producing *E*. *coli* variants on broiler farms. Maximum parsimony trees constructed based on the concatenated sequences of the seven MLST alleles, using Bionumerics 7.1 software. Node sizes reflect the number of isolates per ST and node colours represent different matrices. Additionally indicated are the phylogenetic group, ESBL-genotype and ABR profiles of isolates in every node (i.e., ST). Note that per sample maximally one isolate of each variant was included, and that multiple isolates of a specific variant reflects detection in multiple samples. ABR profiles represent antibiotics to which resistance was observed additionally to 3rd generation cephalosporins. In between brackets are antibiotics with MICs just above and just below epidemiological cut-off values (i.e. with a maximal 2-fold difference) among isolates with the same ST and/or ESBL genotype. Sx = sulfamethoxazole, Tm = trimethoprim; Te = tetracycline, Ci = ciprofloxacin, Na = nalidixic acid, St = streptomycin, Ge = gentamycin, Ax = amoxicillin+clavulanic acid, Ch = chloramphenicol. Br1 was sampled during three production rounds: August/September 2011 (Br1_1), November 2011 (Br1_2), and August/September 2012 (Br1_3); underlined are variants that were detected at multiple time-points.

Inclusion of sequence types (STs), phylogenetic group, ESBL-genotype and antibiotic resistance profiles in variant analysis demonstrated on average 16 and 9.0 different ESBL-producing *E*. *coli* variants at broiler farms and laying hen farms respectively, of which 10.0 and 4.2 were detected in faeces and rinse water (Figs 4 and 5). The diversity among ESBL-producing isolates from flocks was higher on broiler farms (average SID: 0.93) compared to laying hen farms (average SID: 0.63), although this difference was not statistically significant (*P* = 0.071). The lack of statistical significance may at least partially be due to the large variation between laying hen farms and small number of farms analysed. Four of five laying hen farms had SIDs that were considerably lower than for each of the three broiler farms. The only exception was Lh2 which, compared to the other laying hen farms, had an uncharacteristically high diversity of ESBL-producing variants (SID = 0.92, see also Fig 4). Despite the large variation at this latter farm, the variation at the level of antibiotic resistance appeared to be much more limited: eight different STs carried *bla*_CTX-M-1_ in combination with sulfamethoxazole and tetracycline resistance (Fig 4). Indeed, at laying hen farms, a lower diversity was observed at the level of antibiotic resistance properties than at the level of total isolate characteristics (average SIDs 0.37 vs. 0.63, respectively). Although this difference was not statistically significant (*P* = 0.068), it was clearly more pronounced than that at broiler farms (average SIDs 0.90 vs. 0.93, respectively). Based on antibiotic resistance properties alone, the diversity was significantly higher at broiler farms compared to laying hen farms (*P* = 0.036). At laying hen farms, but not at broiler farms, the diversity among ESBL-producing *E*. *coli* from environmental matrices was significantly higher compared to ESBL-producing *E*. *coli* from flocks (Table 3).

### Relation between ESBL-Producing Isolates from Poultry Faeces and Farm Environment

**Fig 6:**
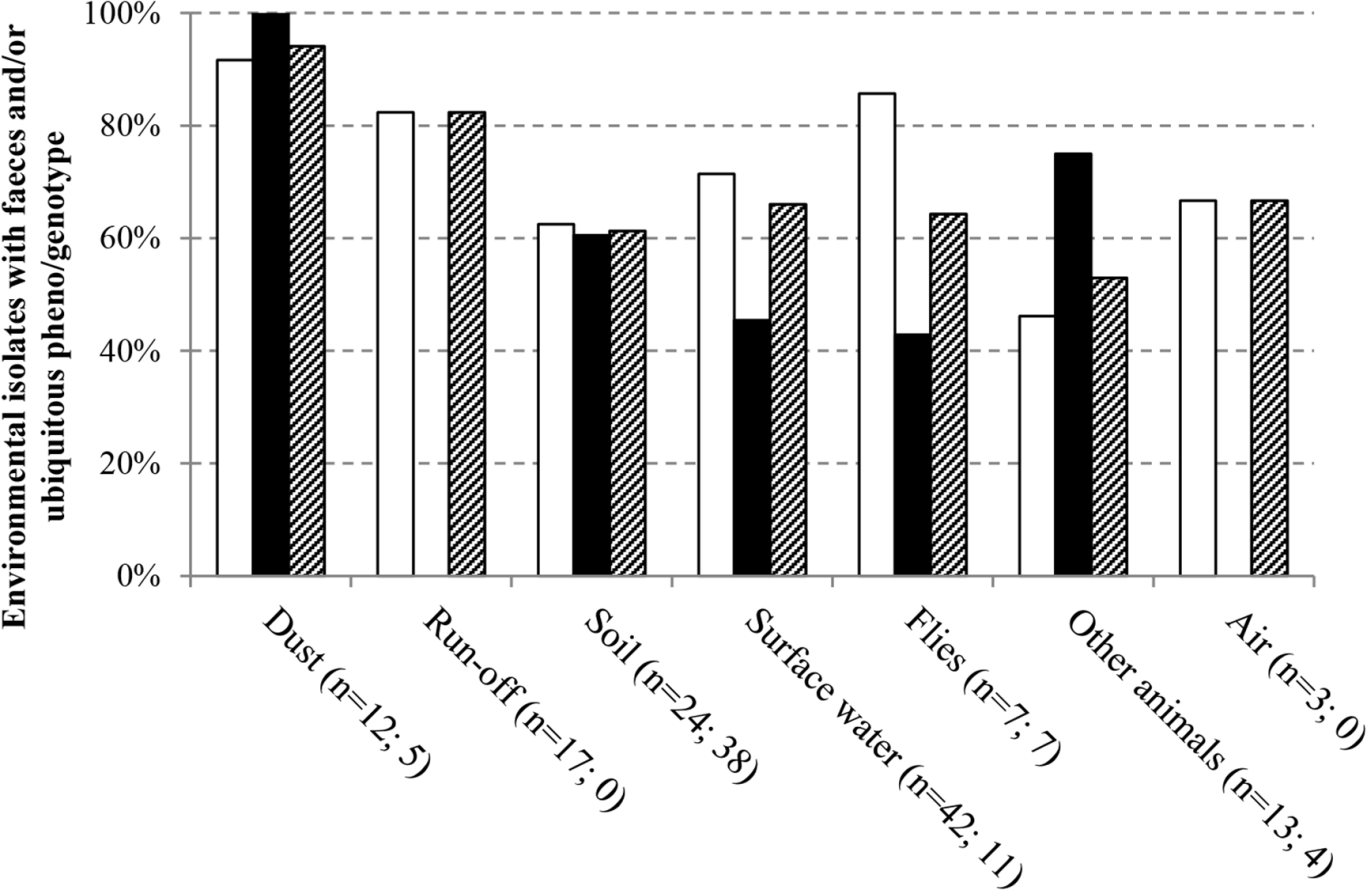
Relationship between ESBL-producing variants from different matrices. Indicated are the proportions of isolates from the different environmental matrices, with identical counterparts in manure and rinse water and/or other environmental matrices at the same farm, based on phylogenetic group, ESBL-genotype, ABR profile and ST. White bars represent broiler farms, black bars represent laying hen farms, and hatched bars represent the results for all farms combined.

Overall, 60% of all isolates from farm environment had a variant type that was identical with respect to ST, phylogenetic group, ESBL-genotype and ABR profile, to variants observed in poultry faeces or rinse water at the same farm (i.e. ‘flock’ variants). An additional 7.1% of isolates from farm environments had identical counterparts in other environmental matrices at the same farm (i.e. ‘ubiquitous’ variants) (Fig 6). The proportion of environmental ESBL-producing isolates with antibiotic properties (i.e. ESBL genotype and ABR profile) identical to those in flocks and/or observed in multiple matrices was 74% and 4.4%, respectively.

The highest proportion of flock and/or ubiquitous variants was observed in dust (94%), run-off gullies (82%) and barn air (67%) (Fig 6). Among surface water, soil and fly variants, 61–66% were identical to those observed in flocks and other environmental matrices; in other farm animals approximately half of the isolates were identical to those in flocks and farm environment. The proportion of flock and ubiquitous variants in the farm environment was slightly higher on broiler (71%) compared to laying hen farms (60%) (*P>0*.*1*). The two farm types differed particularly with respect to the proportions of flock and ubiquitous variants in surface water and on flies (both higher at broiler farms), and that in other farm animals (higher at laying hen farms) (Fig 6), however, none of these differences were statistically significant. Farm animals harbouring ESBL-producing variants identical to those in flocks or in other environmental matrices at the same farm consisted of cattle (Lh3, Br2), swallows (Lh3), dogs (Br1), and hobby laying hens (Br1_1) (Fig 4). An isolate obtained from horse faeces at Lh2 had an ESBL-genotype that was not detected in any of the poultry isolates, *bla*_CTX-M-14_.

At Br1, six of the variants detected at the first time-point (August/September 2011), were still detected a couple of production rounds later, in November of the same year. One of these variants was persistent in hobby laying hens only, three were detected in poultry faeces as well as several environmental matrices at both time points, and two were detected in poultry faeces and farm environment at the first time-point, but only in farm environment (soil and surface water) at the latter time-point (Fig 5). In August/September 2012, the variants present in 2011 were no longer detected.

## Discussion

ESBL-producing *E*. *coli* were detected in flocks at all eight investigated poultry farms, i.e. in broiler and laying hen flocks. A high prevalence of ESBL-producing *E*. *coli* in Dutch broiler flocks has previously been described [13,14], and was shown to be at least partially traceable to breeding chickens and parent stocks [46]. Although a limited number (five) of laying hen farms was included in the present study, our findings demonstrate a high prevalence of ESBL-producing *E*. *coli* in Dutch laying hen flocks as well. The prevalence of ESBL-producing *E*. *coli* in poultry faeces as well as the barn environment (air, dust), was higher at broiler farms compared to laying hen farms. In the Netherlands, the total amount of antimicrobials used in broilers is approximately twenty times higher than that in laying hens: in 2013, 18.1 daily dosages per animal year (dd/ay) were registered for conventionally held broilers, compared to 0.5–1.2 dd/ay for battery and free-range laying hens respectively[47]. The lower use of antibiotics means there is a lower selection pressure on ESBL-producing *E*. *coli* in laying hens as compared with broilers. Assuming that part of the broiler and laying hen flocks already carry ESBL-producing *E*. *coli* at entrance of the production farms, the low selection pressure in laying hens might result in a decrease in ESBL-producing bacteria in flocks over time, while they remain present for a longer time among broilers. This, in combination with the older age of the laying hen flocks compared to the broiler flocks at the time of sampling might explain the observed differences in prevalence.

In the outdoor farm environment, ESBL-producing *E*. *coli* were frequently detected in soil and surface water. Other farm animals when present at the poultry farms, as well as flies, were also shown to carry ESBL-producing *E*. *coli*. Overall, the prevalence in soil was higher at sites that were visibly influenced by poultry faeces, e.g. free-range areas, sites near manure transport belts, near manure storage sheds, near dung heaps and near a rinse water storage container. Both the detection frequency and the average concentrations in soil were higher at laying hen farms compared to broiler farms, which was largely attributable to the inclusion of free-range areas at laying hen farms (which were not present at the broiler farms). Broiler but not laying hen farms significantly contributed to the contamination of surface water, as evidenced from the statistically significant higher prevalence and average concentrations in water adjacent to broiler farms compared to remote sampling sites. This difference appeared largely associated with the cleaning of broiler farms between two production rounds (i.e. every six to seven weeks). The effect of cleaning of barns on the environmental load was not studied at the laying hen farms, due to the long lifespan of laying hens (approximately one and a half year). It is conceivable however, that the observed difference between the two farm types with respect to surface water contamination is mainly associated with the frequency of clearing and cleaning. The high prevalence of ESBL-producing *E*. *coli* in free-range areas suggests that run-off from such areas represents a source of surface water contamination as well. This is supported by the observation that one of the three positive surface water samples obtained in the vicinity of laying hen farms was adjacent to a free-range area. In this water, ESBL-producing *E*. *coli* was detected with the same identity with respect to phylogenetic subgroup, sequence type, ESBL-genotype, and ABR profile, as isolates detected in, amongst others, free-range soil and poultry faeces (Lh3: ST155/B1/CTX-M-1/SxTe). The other two positive surface water sites were in the proximity of a barn ventilation fan (Lh3) and a manure storage shed (Lh4), and also these waters contained isolates that were present in poultry faeces and other environmental matrices at the corresponding farms (Lh3: ST155/B1/CTX-M-1/SxTe, Lh4: ST1463/B1/TEM-52/-).

Comparison of isolates from flocks and farm environment indicated the poultry flocks as major source of environmental contamination. At both types of farms, a very high diversity of ESBL-producing variants with respect to phylogenetic subgroup, sequence type, ESBL-genotype and ABR profile was observed in poultry faeces and barn rinse water (matrices assumed to reflect flock carriage). A small majority (62%) of all variants obtained from the farm environment had the exact same identity as isolates observed in flocks present at the same time at the same farm. It should be emphasised that this percentage presumably reflects an underrepresentation of the proportional load of variants in the farm environment that are identical to ‘flock’ variants. This is the result of sequential rounds of selection of isolates after each test, in which a maximum of one or two isolates with specific characteristics per sample was included for the next round (i.e. selection was aimed to include a maximum of diversity). Based on the current data, the contribution of each variant (including the ‘flock’ variants and the ‘unique’ variants) to the total environmental load cannot be calculated.

The detection of variants in the farm environment not detected in flocks may have multiple explanations. Firstly, part of the variants in the farm environment may originate from other sources than the poultry flocks at the farms. This is most probable for ‘motile’ environmental compartments such as surface water and flies. Surface water, may contain variants introduced upstream of the investigated farms (e.g. from other farms or wastewater treatment plants). Flies may move between habitats [48], and possibly carry ESBL-producing *E*. *coli* from one farm to the next. Other farm animals, when they are not bred at the poultry farms, could also be considered as motile. In the case of other farm animals, the presence of non-poultry isolates might additionally be explained by separate treatment regimens. For barn air and soil on farm premises, the presence of ESBL-producing *E*. *coli* from another origin than the flocks is less easily envisioned. One explanation may be the persistence of bacteria from previous flocks in for instance dust, soil, or water. *E*. *coli* may survive for weeks to months in soil and the aquatic environment, depending on physical, chemical and biological properties of the matrix, such as temperature, pH, sunlight, soil moisture and presence of nutrients [49,50]. This possibility was supported by our own findings at Br1. Following the same line of reasoning, the significantly higher turn-over of flocks at broiler farms as compared to laying hen farms, might contribute to the higher diversity observed on the latter farm type. A second explanation for discrepancies between matrices may simply be chance, related to the probability of omitting variants during the process of colony selection, given the high level of variation and a limited number of selected colonies. In other words, the fact that certain variants were not detected in poultry faeces does not rule out the possibility that they were actually present in faeces. That this may indeed explain at least part of the discrepancies was suggested by the presence of variants in rinse water directly derived from barns, which were not detected in poultry faeces from the same barns (S2 Table). The same explanation might underlie the occasional observation of variants being abundantly present in the farm environment (as indicated by their presence in a variety of different matrices) while not detected in poultry faeces or barn rinse water. A third explanation could be the development of new variants in the environment itself through the exchange of plasmids containing ESBL-genes or other antibiotic resistance genes [17,51–53]. The genes encoding ESBLs are generally located on plasmids that may contain other resistance genes as well [7,8,54,55]. Transfer of mobile elements could explain the observations of isolates with the same ESBL-genotype and/or other ABR profiles in different *E*. *coli* backgrounds (i.e. sequence types) at the same farm. This process may particularly contribute to the variation observed in the other farm animals, assuming intestinal tracts to be an optimal site for horizontal gene transfer [56,57], but possibly also in soil, surface water and sediments of run-off-gullies. *E*. *coli* has been shown to be able to act both as donor and recipient of mobile elements in soil and surface water. The efficiency of the gene transfer among *E*. *coli* in soil was shown to be dependent on its physicochemical properties, such as the presence of nutrients (e.g. amended soil, or non-sterile agricultural soil), temperature (e.g. at 15°C—30°C), and humidity (e.g. at 20%-60%) [58–60]. These conditions may, at least occasionally, be met in soil and surface water at poultry farms. Given the fact that genes responsible for antibiotic resistance are often located on plasmids, the current observation of different STs with identical ESBL-genotype and antibiotic resistance profiles at the same farm, supports the occurrence of horizontal gene transfer. In line with this, the proportion of isolates in the farm environment with antibiotic resistance properties identical to those in the poultry flocks was higher than the proportion of isolates that was exactly the same (74% vs. 62%). Typing of the plasmids that carry ESBL and the other ABR genes could confirm that gene transfer indeed contributes to the diversity observed at farms and in the farm environment [61,62], but this was not part of the aim of the current study.

Our study is one of few studies investigating ESBL producing *E*. *coli* in the natural (i.e. outside) environment at poultry farms [24–28]. The most thoroughly studied exterior environmental matrices so far were ambient air and soil or ground surfaces, whereas surface water was included only once [24]. Altogether, previous and current data demonstrate that dissemination of ESBL-producing *E*. *coli* from livestock to the natural environment occurs. From a public health perspective, the introduction of ESBL-producing *E*. *coli* (and ABR zoonotic bacteria in general) into the environment may pose a risk if these bacteria are transported to places where the general public may become exposed. The environmental compartments that should be considered as likely vehicles for dissemination are water, air, and flies, because of the distance that may be covered (as opposed to e.g. soil). The risk of human exposure through the environment depends on the bacterial concentrations at the source (i.e. farm environment), and the reduction in these concentrations during the travel-time to places where people may become exposed. For exposure through water, factors involved are for example dilution and bacterial die-off in water; human exposure to bacteria carried by flies depends for example on the proportion of flies that migrate from farms, and the numbers of bacteria that survive during this process and are subsequently transferred from flies to food meant for human consumption. The role of water and flies in human exposure to ESBL-producing *E*. *coli* is currently being assessed using quantitative microbial risk assessment (QMRA) (Schijven et al. submitted, Evers et al. in press [63]). Even though our findings did not designate air as a major contaminated compartment at poultry farms, air has previously been implicated as source of dissemination from broiler as well as fattening pig farms [26,28]. However, even though in these studies the prevalence of 3^rd^ generation-resistant *E*. *coli* in barn air was higher (16% in both studies) compared to the current findings (8% at broiler farms), in these studies, the prevalence in ambient air was still relatively low (7.5% and 6%, respectively). In particular surface water may prove to be a relevant route of environmental exposure, because of its use for recreation, irrigation and production of drinking water. Indeed, waterborne transmission has been implicated as an important route of transmission of intestinal bacteria in general [64–66]. Besides animal husbandry farms, other sources likely contribute to the contamination of the aquatic environment, such as arable farming (run-off of animal manure) and discharge of partially treated human wastewater by wastewater treatment plants [18];. To globally reduce the prevalence of ABR bacteria, not only reduced antibiotic use (in humans and animals) but also reduced dissemination of resistant bacteria is important. Further study is required to gain insight in the relative contribution of different contamination sources to the load of clinically relevant ABR bacteria in the (aquatic) environment, which is imperative for the development of effective intervention strategies.

## Supporting Information

S1 Material and Methods Supplemental Material and Methods. (DOCX)

S1 Table Numbers of analysed samples. (DOCX)

S2 Table ESBL-producing *E*. *coli* variants in faeces and barn rinse water at broiler farms. (DOCX)
