# Supplementary material for: Distribution, Numbers, and Diversity of ESBL-Producing E. coli in the Poultry Farm Environment
Source: PLoS One. 2015 Aug 13;10(8):e0135402. doi: 10.1371/journal.pone.0135402 (PMC4536194; doi:10.1371/journal.pone.0135402)
Supplement: S1 Material and Methods — (DOCX) [file pone.0135402.s001.docx]

**Supplemental Material and Methods.**

*Faeces*

Samples of poultry faeces consisted of five individual droppings, with the exception of the samples from dung heaps and storage containers or storage sheds of which 20 - 50 grams were collected in sterile containers. Samples of faeces from other farm animals (cattle, horses, dog, swallows) consisted of material from individual droppings. All faeces samples were placed in sterile containers. Samples were pre-homogenized by addition of BPW in a 1:1 or 1:2 ratio and using a sterile spoon. Equivalents of 10 g of faeces were next suspended in 90 ml of buffered peptone water (BPW) and homogenized using a Pulsifier® (Microgen Bioproducts, Camberley, UK). From the homogenate ten-fold serial dilutions were prepared in pepton-physiological salt (PPS) and 100 µl of different dilutions was streaked in two-fold onto tryptone bile X-glucuronide (TBX) agar (10^-1^ to 10^-6^) and ChromID® ESBL agar (10^-1^ and 10^-2^). Additionally, a sample of the pre-homogenized sample (diluted 1:1 or 1:2) was swabbed onto ChromID®ESBL agar using a sterile cotton swab. To the remainder of the homogenate (equivalent ~10 g manure), 1μg/ml cefotaxime (CTX) was added for enrichment of ESBL-producing *E. coli.*

*Soil and sediment*

Soil was sampled from the surface to a depth of 5 to 8 cm, using a tubular soil sampler. At each site, 9 to 12 grab samples were obtained that were evenly distributed over a rectangular or square area with in-between distances of 0.5 to 1 m. The 9 to 12 grab samples from a site were pooled in sterile filter bags (BagPage®, Interscience, St. Nom la Bretêche, France), resulting in 60-90 g of pooled soil samples. Sediments from (nearly) dried-up run-off gullies were sampled using a sterile spoon and collected in sterile containers. Soil and sediment samples were diluted 1:1 in BPW, and homogenized using a Pulsifier® (Microgen Bioproducts, Camberley, UK). From the homogenate ten-fold dilution series were prepared in pepton-physiological salt (PPS) and 100 µl of different dilutions was streaked in two-fold onto tryptone bile X-glucuronide (TBX) agar (1:1, 10^-1^ , 10^-2^) and ChromID® ESBL agar (1:1). Equivalents of 10g of soil were enriched in 90ml of BPW supplemented with 1µg/ml CTX.

*Surface water and rinse water*

Surface water and wastewater was sampled by submerging sterile glass bottles, according to NEN-EN-ISO 19458. From each sample, multiple volumes were filtered through 0.45 μm pore size membrane filters (Millipore, Amsterdam, the Netherlands). Volumes ranged from 0.1 to 200 ml, depending on the target- organism (*E. coli* or ESBL-producing *E. coli*) and type of water (surface water or rinse water). The maximal volume of surface water analysed for the presence of ESBL-producing *E. coli* per sample, varied between 172 and 720 ml (depending on the turbidity of the water, as well as the sampling year), corresponding to a lower detection limit of 1 to 6 cfu/l.

*Dust*

Dust was sampled from surfaces using sterile sponges (Meat and Turkey Carcass Sampling Kit, Nasco Canada) that were pre-soaked with BPW, or by scooping it up in sterile containers. After sampling sponges were placed in sterile bags for transport to the laboratory, where they were submerged in 90 ml BPW either or not supplemented with 1µg/ml CTX. For quantification, 5 to 10g of dust was added to 90 ml BPW, homogenized using a Pulsifier®, and serial diluted in PPS. Of different dilutions 100 µl was streaked onto TBX agar (10^-2^ to 10^-6^) and ChromID® ESBL agar (10^-1^ to 10^-4^) in two-fold. The remainder of the homogenate was enriched in BPW+1µg CTX.

*Air*

Air was sampled through 8 μm pore size membrane filters using a Sartorius MD8 Airport (Sartorius Stedim Biotech, Nieuwegein, the Netherlands), or alternatively, by placing TBX agar- and ChromID®ESBL agar-containing plates without lids on top of heat convectors that were suspended approximately 1 m above the stable floors, for 50 to 75 minutes. Using the Sartorius Airport, 250 or 500 L of air was sampled at 40 or 50L/min. Upon sampling, membrane filters were directly submerged in 180 ml BPW.

*Flies*

Flies were collected using non-toxic, sticky flypaper, and harvested within 24 hours after placement. When the number of flies stuck on the flypaper was considered low compared to the number of flies flying around at the moment of ‘harvest’, they were additionally collected using a fly swatter and stored in sterile containers. At the broiler farms 117 flies and at the laying hen farms 209 flies were collected. These 326 flies were analysed in 73 separate pools, each pool consisting of one to eight flies that were identical with respect to collection site and fly species (or when applicable with respect to genus or fly family). The collected flies consisted of 212 *M. domesticus* or common house flies (32 samples), 39 *Stomoxys calcitrans* or stable flies (11 samples), 26 *Fannia canicularis* or lesser houseflies (9 samples), 20 flies belonging to the family of *Calliphoridae* or blowlies (8 samples), 10 *Muscina stabulans* or false stable flies (6 samples), 11 flies belonging to the genus Hydrotaea (4 samples), 6 flies belonging to the family of *Tachinidae* (2 samples), and 2 flies belonging to the family of *Sarcophagidae* or flesh flies (1 sample). Using sterile pairs of tweezers flies were transferred to sterile filter bags (Interscience, St Nom La Bretêche, France) containing 24 to 33 ml phosphate-buffered saline (Biotrading, Mijdrecht, The Netherlands) with 0.5% Tween-20. Flies were thoroughly crushed using thumb and forefinger (from the outside of the bag) and then homogenized, using a Stomacher®400 (Seward, Worthing, UK) at 230 rpm. Of these homogenates, 100 µl was streaked in two-fold on ChromID^TM^ ESBL medium (Biomerieux, Boxtel, the Netherlands), and 100ul of 10^-1^ ad 10^-2^ dilutions in PPS were streaked in two-fold onto TBX. Additionally, 10 ml of the homogenates was pre-enriched in BPW either or not supplemented with 1 µg/ml cefotaxime (BPW/CTX).
