# Supplementary material for: Distribution, Numbers, and Diversity of ESBL-Producing E. coli in the Poultry Farm Environment
Source: PLoS One. 2015 Aug 13;10(8):e0135402. doi: 10.1371/journal.pone.0135402 (PMC4536194; doi:10.1371/journal.pone.0135402)
Supplement: S1 Table — (DOCX) [file pone.0135402.s002.docx]

Table S1. Numbers of analysed samples.

|  | Br1_1 | Br1_2 | Br1_3 | Br2 | Br3 | Lh1 | Lh2 | Lh3 | Lh4 | Lh5 | Tot |
| --- | --- | --- | --- | --- | --- | --- | --- | --- | --- | --- | --- |
| Poultry faeces (belts) | 0 | 0 | 0 | 0 | 0 | 2 | 0 | 2 | 2 | 5 | 11 |
| Poultry faeces (droppings) | 15 | 8 | 8 | 15 | 8 | 9 | 12 | 9 | 8 | 8 | 100 |
| Poultry faeces (storage) | 2 | 2 | 2 | 4 | 6 | 4 | 4 | 2 | 3 | 1 | 30 |
| Rinse water | 5 | 5 | 2 | 3 | 1 | 0 | 0 | 1 | 0 | 0 | 17 |
| Run-off gullies^a^ | 3 | 3 | 1 | 0 | 2 | 0 | 0 | 0 | 0 | 0 | 9 |
| Other animals^b^ | 2 | 1 | 3 | 4 | 0 | 0 | 1 | 3 | 0 | 0 | 14 |
| Soil | 10 | 13 | 6 | 8 | 11 | 8 | 9 | 8 | 4 | 10 | 87 |
| Surface water (<50m) | 3 | 5 | 7 | 9 | 3 | 2 | 0 | 2 | 4 | 0 | 35 |
| Surface water (>50m) | 0 | 1 | 8 | 1 | 7 | 2 | 2 | 2 | 0 | 0 | 23 |
| Dust (inside) | 12 | 4 | 0 | 0 | 0 | 0 | 0 | 0 | 9 | 6 | 31 |
| Dust (outside) | 3 | 1 | 0 | 0 | 0 | 0 | 0 | 0 | 0 | 0 | 4 |
| Air (inside) | 4 | 6 | 16 | 0 | 0 | 0 | 0 | 0 | 4 | 3 | 33 |
| Air (outside) | 4 | 0 | 0 | 0 | 0 | 0 | 0 | 0 | 0 | 0 | 4 |
| Flies | 11 | 1 | 9 | 5 | 3 | 17 | 6 | 9 | 4 | 8 | 73 |
| Total | 74 | 50 | 62 | 49 | 41 | 44 | 34 | 38 | 38 | 41 | 471 |

Br1 – Br3 = Broiler farm 1 to 3, Lh1 – Lh5 = Laying hen farm 1 to 5; at laying farms samples were obtained from two visits (‘orienting’ and ‘main’), at broiler farms Br2 and Br3 from three visits (‘orienting’, ‘main’, ‘during/after cleaning’). Broiler farm Br1 was sampled during three different production rounds (Br1_1 to Br1_3). Br1_1 to Br1_3 all included samples obtained during the ‘main’ visit and ‘during/after cleaning’; samples obtained at the orienting visit were included in Br1_1. ^a^Water, or sediment when gullies were dried-up; ^b^Faeces from other livestock, companion animals or wild birds.
