# Supplementary material for: Distribution, Numbers, and Diversity of ESBL-Producing E. coli in the Poultry Farm Environment
Source: PLoS One. 2015 Aug 13;10(8):e0135402. doi: 10.1371/journal.pone.0135402 (PMC4536194; doi:10.1371/journal.pone.0135402)
Supplement: S2 Table — (DOCX) [file pone.0135402.s003.docx]

Table S3. ESBL-producing *E. coli* variants in faeces and barn rinse water at broiler farms.

| Variant type | No. isolates detected in: | | Total no. of flock variants |
| --- | --- | --- | --- |
|  | **Poultry faeces** | **Rinse water from barns** |  |
| Br1_1 |  |  |  |
| ST3519/A0/SHV-12/SxTe(St)Ch | 2 | 1 | 3 |
| ST10/A1/SHV-12/SxTe(St)Ch | 2 | 0 | 2 |
| ST420/D2/SHV-12/CiNa | 1 | 0 | 1 |
| ST448/B1/CTX-M-1/SxTmCiNaSt(Ax) | 2 | 0 | 2 |
| ST2079/B1/SHV-12/SxTmTeCiNaStCh | 0 | 1 | 1 |
| ST155/B1/SHV-12/SxTe(St)Ch | 0 | 2 | 2 |
| ST58/B1/SHV-12/Ci(Ax) | 1 | 3 | 4 |
| ST212/B1/SHV-12/SxTe(St)Ch | 0 | 1 | 1 |
| ST4994/B22/TEM-52/SxTmCiNa | 1 | 0 | 1 |
| Br1_2 |  |  |  |
| ST10/A1/SHV-12/SxTe(St)Ch | 2 | 1 | 3 |
| ST1844/B1/CTX-M-1/SxTmTeSt | 1 | 0 | 1 |
| ST117/D2/CTX-M-1/TeCiNa | 1 | 1 | 2 |
| ST683/B1/SHV-12/SxTmTeCiNaStCh | 1 | 0 | 1 |
| ST2309/D1/CTX-M-1/SxTmTe | 1 | 0 | 1 |
| ST155/B1/SHV-12/SxTe(St)Ch | 2 | 0 | 2 |
| ST10/A1/SHV-12/SxTmTeCh | 0 | 1 | 1 |
| ST4994/B22/TEM-52/SxTmCiNa | 2 | 0 | 2 |
| ST4980/A0/SHV-12/SxTmTeStCh | 1 | 0 | 1 |
| Br1_1 |  |  |  |
| ST997/D1/CTX-M-1/SxTmTeCiNa | 1 | 0 | 1 |
| ST1844/B1/TEM-52/SxTmTeSt(Ax) | 1 | 1 | 2 |
| ST997/D1/CTX-M-1/SxTeCiNa | 2 | 1 | 3 |
| ST57/D2/CTX-M-1/SxTmTeCiNaCh | 2 | 0 | 2 |
| ST616/B1/SHV-12/TeCiSt | 1 | 1 | 2 |
| ST2309/D1/SHV-12/TeCi | 1 | 0 | 1 |
| Br2 |  |  |  |
| ST10/A1/CTX-M-1/SxTeCiNa | 2 | 1 | 3 |
| ST1158/D1/TEM-52/SxTeCiNaSt | 1 | 0 | 1 |
| ST155/B1/TEM-52/SxTmTeCiNaSt | 2 | 1 | 3 |
| ST1594/A0/CTX-M-1/SxTe | 0 | 1 | 1 |
| ST1594/A0/CTX-M-1/SxTmTeSt | 1 | 0 | 1 |
| ST1610/B1/CTX-M-1/- | 1 | 0 | 1 |
| ST1684/A0/TEM-52/- | 0 | 1 | 1 |
| Br3 |  |  |  |
| ST117/D2/SHV-12/SxTeStCh | 1 | 0 | 1 |
| ST117/D2/SHV-12/SxTeCiNaCh | 1 | 0 | 1 |
| ST1564 /A0/CTX-M-1/SxTmTeSt | 1 | 1 | 2 |
| ST1818/A0/TEM-52/SxTmTeCiNaSt | 1 | 0 | 1 |
| ST189/A0/TEM-52/SxTmTeCiNaStCh | 1 | 1 | 2 |
| ST219/B22/CTX-M-1/SxTmSt | 0 | 1 | 1 |
| ST295/B1/SHV-12/SxTmTeSt(Ax)Ch | 3 | 0 | 3 |
| ST371/D2/CTX-M-1/SxTm | 1 | 0 | 1 |
| ST420/D2/SHV-12/SxTmTeCiNaSt | 1 | 0 | 1 |
| ST1818/D1/TEM-52/SxTmTeCiNaSt | 1 | 0 | 1 |
